# In vitro infection efficiency of nervous necrosis virus alters depending on amount of viral particles adsorbed onto cells

**DOI:** 10.1038/s41598-023-39426-6

**Published:** 2023-07-29

**Authors:** Han Sol Lee, Hyun Jung Gye, Toyohiko Nishizawa

**Affiliations:** 1 Department of Aqualife Medicine, Chonnam National University, Yeosu, 59626 Republic of Korea; 2 Present Address: West Sea Fisheries Research Institute, National Institute of Fisheries Science, Incheon, 22383 Republic of Korea

**Keywords:** Virus-host interactions, Infectious diseases, Viral infection, Mechanisms of disease

## Abstract

Nervous necrosis virus (NNV) in the family *Nodaviridae* is one of the simplest spherical RNA viruses and is pathogenic to many fish species. We investigated the effect of purified NNV on striped snakehead cells (SSN-1) in terms of adsorption ratio and infection efficiency using the 96-well titration system. The proportion of cytopathic effect (CPE)-positive wells among total number of wells inoculated with the virus (CPE appearance ratio) reduced by 17% each time the NNV infectivity dose was halved (*y* = *55.7x* + *50.6*). Thus, subtle differences in NNV infectivity could be accurately detected using this system. Experiments performed to observe alteration of CPE appearance ratio with changing viral doses and adsorption times showed that NNV particles introduced into microplate wells as suspensions in ≤ 100 µl inoculum were adsorbed almost completely onto cells seeded on the wells within 4 days of incubation. Density profile analysis of NNV coat proteins revealed that the NNV suspension at 1 50% tissue culture infectious dose (TCID_50_) contained 60 particles. Infection efficiency/NNV peaked at 20 particles (1.20%/particle) and then declined gradually with increasing NNV doses. Therefore, in vitro infection efficiency of NNV may alter depending on the quantity of viral particles adsorbed onto cells.

## Introduction

Nervous necrosis virus (NNV) belonging to the *Betanodavirus* genus in the *Nodaviridae* family is one of the simplest spherical RNA viruses^1^ that is highly pathogenic to more than 170 fish species worldwide^2–4^. NNVs are non-enveloped spherically shaped particles approximately 25–30 nm in diameter that consist of a single coat protein (CP, *M*_r_ 42,000) and two molecules of positive-sense single-stranded RNA^1^. Advances in in vitro studies regarding NNV occurred since the successful cultivation of NNV using the SSN-1 cell line, which was established during the 1990s from the striped snakehead fish, *Channa striata*^5^. Subsequently, several primary cell lines susceptible to NNV have been established using different fish species. However, considering widely distributed cell lines are limited^6^, the SSN-1 cell line is still used to evaluate NNV infectivity.

The entry of NNV particles has been reported to occur via clathrin-mediated endocytosis^7,8^. Certain cell membrane proteins, such as grouper heat shock cognate protein 70 (GHSC70)^9^, marine medaka heat shock protein 90ab1 (MmHSP90ab1)^8^ and nectin-4^10^, function as receptors or co-receptors for the attachment of NNV particles. NNV is also known to utilize sialylated *N*-linked glycans (*N*-glycans) on the surface of SSN-1 cells^11^. Recently, pocket structures at the apex of surface protrusions on NNV particles were shown to be the sites responsible for binding to N-glycan antennae with Neu5Ac-Gal-GlcNAc terminal moieties (N-acetylneuraminic acid, galactose and N-acetylglucosamine)^12,13^.

A better understanding of NNV infection mechanisms requires detailed quantitative analysis of NNV adsorption and infection. Viral particle-to-plaque forming unit (PFU) ratios in cell cultures generally vary depending on the virus species ^14^. NNV infectivity is usually titrated using a 96-well microtiter plate method seeded with SSN-1 cell based on 50% tissue culture infectious dose (TCID_50_) because plaque contours formed by NNV infection on SSN-1 cell monolayer are unclear. Therefore, despite NNV’s simple structure, the ratio of NNV particles adsorbed onto cells and quantity of adsorbed particles that cause infection have not been elucidated, even in vitro.

In this study, we sought to accurately verify the subtle differences in NNV infectivity by titration. The ratio of NNV particles adsorbed onto SSN-1 cells seeded on 96-well microplates was investigated. Subsequently, the number of NNV particles in 1 TCID_50_ was calculated by evaluating the density profile of highly purified NNV and its infectious dose. Based on these results, we aimed to predict the infection efficiency of NNV particles that were adsorbed onto cells in vitro.

## Results

### Correlation between NNV infectivity and cytopathic effect (CPE) appearance ratio

**Figure 1:**
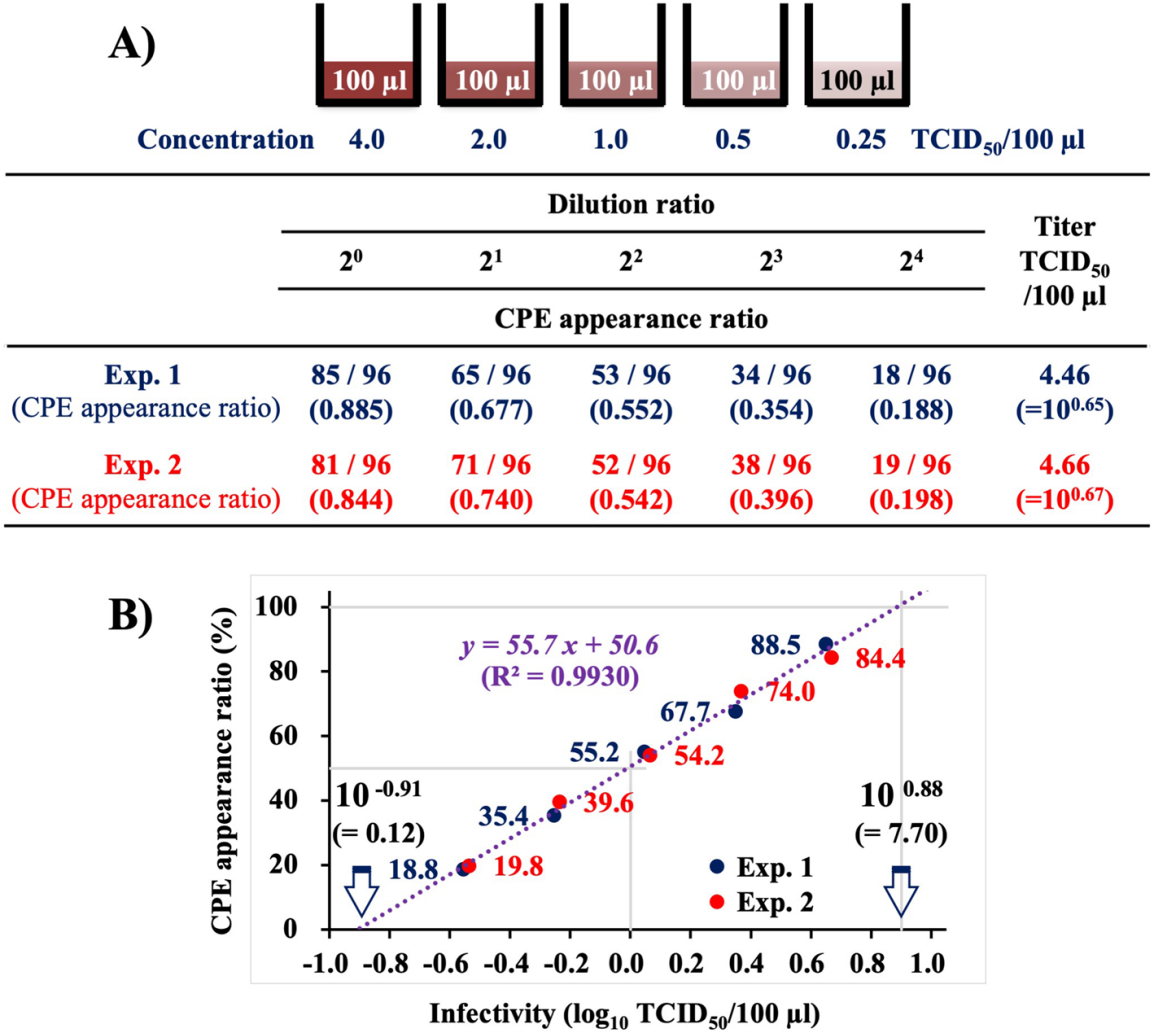
Correlation of NNV infectivity with CPE appearance ratio demonstrated by titration using the 96-well system. (**A**) CPE appearance ratio in serially two-fold diluted NNV at approximately 4 TCID_50_/100 µl. The experiment was replicated twice. (**B**) Semilogarithmic graph showing CPE appearance ratio (%) on the Y axis and the NNV infectivity titer (log_10_ TCID_50_/100 µl) on the X axis. NNV, nervous necrosis virus; CPE, cytopathic effect; TCID_50_, 50% tissue culture infectious dose.

Purified NNV suspension of approximately 4.0 TCID_50_/100 µl was diluted serially twice, and infectivity titration was conducted using the 96-well system (Fig. 1). The infectivity titer of the NNV suspension calculated using modified Kärber’s numerical formula^15^ based on the CPE appearance ratio at each dilution was 4.56 TCID_50_/100 µl, which was the average of two replicates of the same experiment (4.46 and 4.66 TCID_50_/100 µl) (Fig. 1A). The CPE appearance ratio refers to the proportion of CPE-positive wells among total number of wells inoculated with the virus at each individual dilution, which indicates the probability of infection due to inoculated NNV. When these data were plotted on a semilogarithmic graph with CPE appearance ratio (%) on the Y axis and NNV infectivity (log_10_ TCID_50_/100 µl) on the X axis (Fig. 1B), a regression line (*y* = *55.7x* + *50.6*, R^2^ = 0.9930), demonstrating that CPE appearance ratio is closely related to NNV infectivity titer, was obtained. This regression line indicated that the CPE appearance ratio reduced by 17% (= 55.7 × log_10_ 0.5) each time the NNV infectivity dose was halved. Thereafter, infectivity titers of NNV were calculated using the 96-well system based on this regression line.

### Effect of NNV inoculum volume in CPE appearance ratio

**Figure 2:**
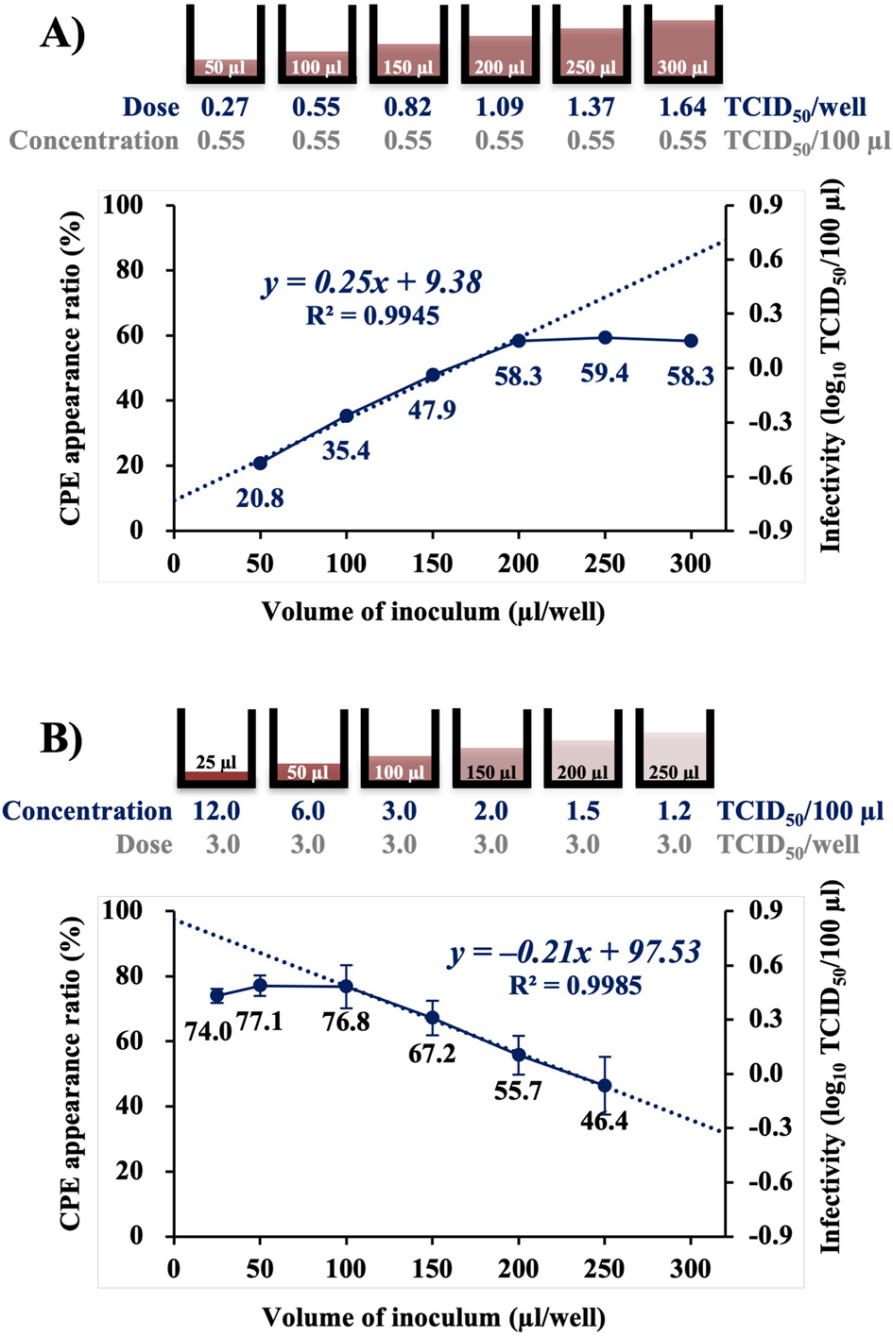
Effect of NNV inoculum volume in CPE appearance ratio. (**A**) Alteration in CPE appearance ratio by inoculating with NNV samples of different volumes and same concentration. Varying volumes (50, 100, 150, 200, 250 and 300 µl) of NNV suspension at 0.55 TCID_50_/100 µl were inoculated onto SSN-1 cells seeded on 96-well microplates. (**B**) Alteration in CPE appearance ratio by inoculating with NNV samples of different volumes and constant dose. NNV suspensions at 12, 6.0, 3.0, 2.0, 1.5 and 1.2 TCID_50_/100 µl were prepared and inoculated to SSN-1 cells seeded on 96-well microplates each by 25, 50, 100, 150, 200 and 250 µl, respectively. Therefore, NNV inoculum dose was constantly 3.0 TCID_50_/well. CPE appearance ration was observed after incubation at 25 °C for 2 weeks. NNV, nervous necrosis virus; CPE, cytopathic effect; TCID_50_, 50% tissue culture infectious dose.

Alteration in CPE appearance ratios due to inoculation with NNV samples of varying volumes and same concentrations were investigated. NNV suspension at 0.55 TCID_50_/100 µl was inoculated to SSN-1 cells seeded on 96-well plates. Six volumes (50, 100, 150, 200, 250 and 300 µl) were introduced, leading to a total of 576 wells (96 wells × 6 volumes). The inoculated cells were incubated at 25 °C for 2 weeks. This experiment was designed to determine whether the probability of establishing infection depends on the NNV concentration or dose. The CPE appearance ratio increased with increasing NNV volume up to 200 µl/well, beyond which the increase was negligible (Fig. 2A).

In the next experiment, alterations in CPE appearance ratio based on inoculation with NNV samples of different volumes and the same dose were investigated (Fig. 2B). NNV suspensions at 12, 6.0, 3.0, 2.0, 1.5 and 1.2 TCID_50_/100 µl were inoculated to SSN-1 cells seeded on 96-well plates each at 25, 50, 100, 150, 200 and 250 µl, respectively. Therefore, the NNV inoculum dose was constantly 3.0 TCID_50_/well. In this experiment, alteration in the probability for establishment of infection was assumed because the concentration of NNV was continuously enriched. The inoculated cells were incubated at 25 °C for 2 weeks. The CPE appearance ratio increased linearly with decreasing NNV volume from 250 µl to 100 µl (corresponding concentration increased from 1.2 to 3.0 TCID_50_/100 µl); however, it did not increase thereafter despite further reduction in volume from 100 µl to 25 µl (corresponding concentration increased from 3.0 to 12.0 TCID_50_/100 µl) (Fig. 2B).

### Correlation between CPE appearance ratio and NNV adsorption times

**Figure 3:**
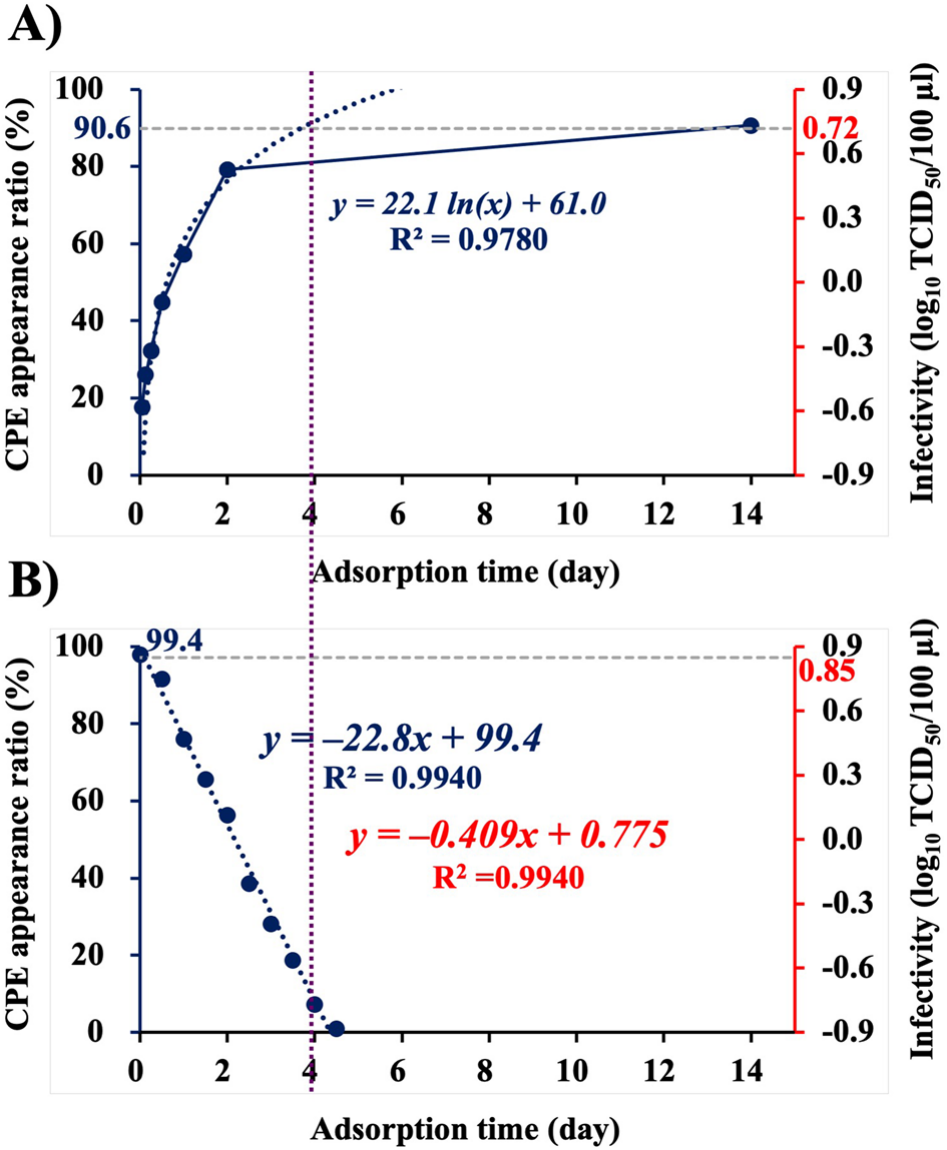
Alteration of CPE appearance ratio and infectivity of NNV depending on adsorption times. (**A**) Alteration of CPE appearance ratio depending on NNV-adsorption time. NNV at 5.25 TCID_50_/100 µl was inoculated and adsorption after 1, 3, 6, 12, 24 and 48 h and then after 2 weeks with and without washing was observed. (**B**) Infectivity alteration of NNV that remained in culture supernatant depending on NNV adsorption time. NNV at 10^1.7^ TCID_50_/100 µl was inoculated to SSN-1 cells seeded on 96-well microplates. After every 12 h of adsorption, the NNV inoculum was harvested and inoculated to fresh cells to measure infectivity of NNV that remained in culture supernatant. NNV, nervous necrosis virus; CPE, cytopathic effect; TCID_50_, 50% tissue culture infectious dose.

Alteration of CPE appearance ratio depending on NNV adsorption times was investigated (Fig. 3A). NNV at 5.25 (= 10^0.72^) TCID_50_/100 µl was introduced in seven 96-well microplates seeded with SSN-1 cells. After 1, 3, 6, 12, 24 and 48 h of incubation at 25 °C, the NNV particles in the wells of six microplates were removed by washing the cells with L-15 medium five times. Subsequently, L-15_10_ medium was added at the rate of 100 µl/well each, and the cells were incubated at 25 °C for 2 weeks. The remaining microplate was incubated for 2 weeks without removing the NNV inoculum (Fig. S1A). This experiment was designed to observe alteration in the probability of establishing infection depending on adsorption times of NNV. The time course alteration of CPE appearance ratio is shown in a graph with appearance ratio (%) on the 1st Y axis, infectivity (log_10_ TCID_50_/100 µl) on the 2nd Y axis, and adsorption time on the X axis (Fig. 3A). The CPE appearance ratio increased with increasing adsorption time up to day 2 and reached close to 80% (a regression line: *y* = *22.1 ln[x]* + *61.0,* R^2^ = 0.9780). After 14 days of adsorption, it was 90.6% (corresponding to 10^0.72^ TCID_50_), which intersected with the regression line at 3.8 days (Fig. 3A). Therefore, the CPE appearance ratio reached the maximum level after 4 days of NNV adsorption (Fig. 3A).

In the next experiment, time course alteration in infectivity of NNV that remained in the culture supernatant depending on adsorption times was investigated (Fig. 3B). NNV at 10^1.7^ TCID_50_/100 µl was inoculated into 96 wells seeded with SSN-1 cells and incubated at 25 °C for 12 h. The NNV inoculum was harvested and mixed for inoculation onto SSN-1 cells seeded on 96-well microplates. After removing the NNV inoculum from all the wells, fresh L-15_10_ medium was added at the rate of 100 µl/well and incubated at 25 °C for an additional 2 weeks. This procedure was repeated 10 times every 12 h (Fig. S1B). This experiment was designed to study alteration of the dose of NNV remaining in the culture fluid depending on progressing adsorption time. The CPE appearance ratio decreased lineally with increasing adsorption times, thus producing the following regression line: *y* = *–22.8x* + *99.4*, R^2^ = 0.9940 (Fig. 3B), which could be transformed into another one relative to the 2nd Y axis as follows: *y* = *–0.41x* + *0.78* because the CPE appearance ratio correlated precisely with the logarithm of NNV infectious doses (Fig. 3B). This function indicates a decrease of 10^–0.41^ (= 0.39) TCID_50_ every 24 h of adsorption; i.e., approximately 98% of NNV was adsorbed onto the cells within 4 days.

### Number of NNV particles in 1 TCID_50_

**Figure 4:**
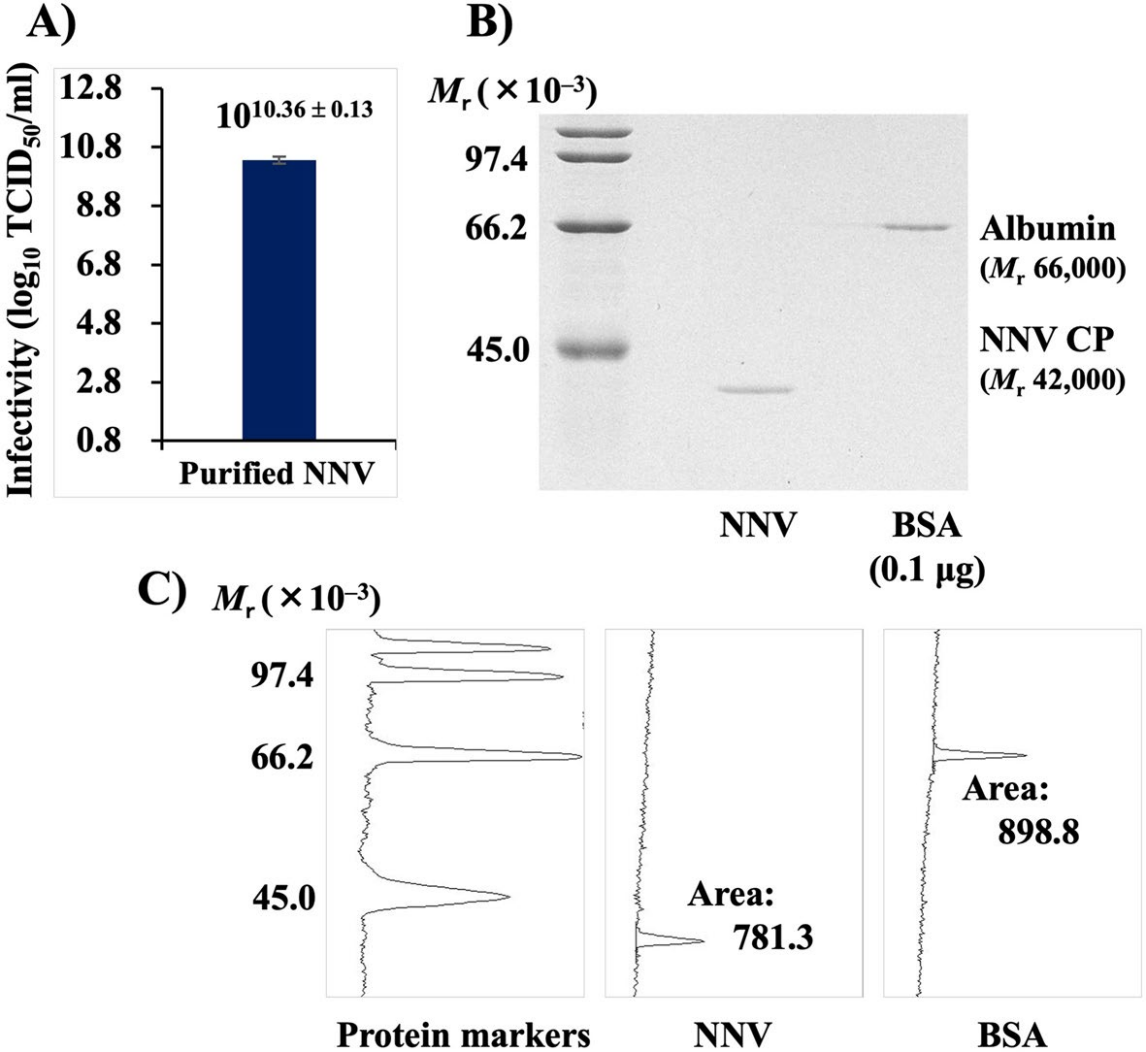
Density profile of digital images of NNV CPs in SDS–polyacrylamide gel. (**A**) Infectivity titer of purified NNV suspension. (**B**) SDS–polyacrylamide gel electrophoresis of purified NNV suspension and BSA. Gel proteins were stained with CBB. NNV suspension and BSA solution (20 µg/ml) were loaded at the rate of 5 µl/lane. An untrimmed photograph of this gel is shown in Fig. S3. (**C**) Density profiles for digital images of NNV CP, BSA and protein markers. Areas of peaks for NNV CP and BSA (20 µg/ml) were 781.3 and 898.8, respectively. NNV, nervous necrosis virus; CP, coat protein; SDS, sodium dodecyl sulfate; BSA, bovine serum albumin; CBB, Coomassie brilliant blue.

Infectivity titer of the highly purified NNV particle suspension was 10^10.36±0.13^ TCID_50_/ml (Fig. 4A). To estimate protein concentration of NNV, sodium dodecyl sulfate–polyacrylamide gel electrophoresis (SDS-PAGE) of purified NNV and bovine serum albumin (BSA) at 20 µg/ ml was conducted. Coomassie brilliant blue (CBB) staining of the gel proteins showed single bands of NNV CP with *M*_r_ 42,000 and BSA with *M*_r_ 66,000 in each sample (Fig. 4B). Density profiles and digital images of NNV CP, BSA and protein markers in the gel are shown in Fig. 4C. Based on the area ratio of peaks of NNV CP and BSA (20 µg/ml), which was 781:899, the NNV CP concentration in the purified NNV suspension was calculated as follows: 20 × 781/899 = 17.4 µg/ml. Molecular mass of NNV particles is 7.56 × 10^6^ because an NNV particle is composed of 180 molecules with CPs having *M*_r_ 42,000^1^. The number of virus particles was calculated using the following formula:

$${\text{Number}}\;{\text{of}}\;{\text{particles}} = \frac{{\left( {{\text{Amount}}\;{\text{of}}\;{\text{substance}}} \right) \times \left( {{\text{Avogadro}}\;{\text{constant}}} \right)}}{{\left( {{\text{Molecular}}\;{\text{mass}}\;{\text{of}}\;{\text{substance}}} \right)}}$$

The NNV suspension (17.4 × 10^–6^ g/ml) contained 1.39 × 10^12^ particles/ml, which was calculated as follows: (17.4 × 10^–6^) × (6.02 × 10^23^)/(7.56 × 10^6^) = 1.39 × 10^12^. The infectivity titer of this suspension was 2.30 × 10^10^ (= 10^10.36±0.13^) TCID_50_/ml (Fig. 4A). Therefore, number of NNV particles in 1 TCID_50_ was calculated as follows: (1.39 × 10^12^ g/ml)/(2.30 × 10^10^) = 60 particles.

## Discussion

Titration of purified NNV using the 96-well system showed that CPE appearance ratio is closely related to logarithm of NNV infectivity titer (regression line: *y* = *55.7x* + *50.6,* R^2^ = 0.9930) (Fig. 1B). The virus dose that demonstrated CPE in 50% of the inoculated wells was defined as 1 TCID_50_, and the CPE appearance ratio at 1 TCID_50_ calculated from the resulting regression line was 50.6%, which deviated from the theoretical value (50%) by 0.6%. Therefore, titration using the 96-well system can accurately detect subtle differences in NNV infectivity. This regression line also indicated that each time the NNV infectivity doses was halved, the CPE appearance ratio reduced by 17%. The intersection of the regression line with the X axis was at 10^–0.91^ (= 0.12) TCID_50_/100 µl, whereas that with *y* = *100* was at 10^0.88^ (= 7.7) TCID_50_/100 µl (Fig. 1B), suggesting that CPE does not appear in wells inoculated with ≤ 10^–0.91^ (= ≤ 0.12) TCID_50_/100 µl of NNV, while it does in all wells inoculated with ≥ 10^0.88^ (= ≥ 7.7) TCID_50_/100 µl of NNV.

The experiments to show the effect of NNV inoculum volume on CPE appearance ratio revealed that CPE appearance ratio increased with increasing NNV volume up to 200 µl/well; thereafter, the increase was negligible (Fig. 2A). These results suggest that the NNV infection efficiency decreases significantly when the inoculum volume is ≥ 200 µl/well. Furthermore, the experiment to determine alteration of CPE appearance ratio due to changes in NNV enrichment (Fig. 2B) showed a strong correlation between CPE appearance ratio and NNV concentration when the inoculum volume was ≥ 100 µl/well. However, when this volume was ≤ 100 µl, CPE appearance ratio did not increase with increasing NNV concentration (Fig. 2B). These results suggest that almost all the NNV particles suspended in ≤ 100 μl inoculum are adsorbed onto the cells seeded on microplate wells. To prove this hypothesis, the correlation between CPE appearance ratio and NNV adsorption time was analyzed (Fig. 3). The CPE appearance ratio increased drastically and reached the maximum level within 4 days of adsorption (Fig. 3A). Moreover, the NNV particles lost approximately 98% of their infectivity within 4 days of adsorption onto the cells (Fig. 3B). Measuring the amount of NNV adsorbed onto the cells or that which remained in the culture supernatant led to the same conclusion; i.e., almost all NNV particles in the culture supernatant were adsorbed onto cells within 4 days. There may be a large difference in the time taken for cell adsorption and that for cell infection between NNV present closer and farther from the cells. Therefore, most of the NNV particles introduced into 96-well microplates as suspensions in ≤ 100 µl might have been adsorbed onto cells.

To evaluate the efficiency with which NNV particles infected SSN-1 cells in vitro, density profile of NNV CPs in a polyacrylamide gel was analyzed (Fig. 4). NNV suspension at 1 TCID_50_ contained 60 particles. Multiplying the TCID_50_ titer (per ml) by 0.69 (= − *ln [0.5]*) yielded the particle-to-plaque ratio of NNV (60 × 0.69 = 41.5), which helped predict the mean number of PFU/ml. This value (41.5), though relatively low, was within the range of values previously reported for various viruses^14^. We found that all NNV particles suspended in ≤ 100 µl of inoculum get adsorbed onto the cells seeded on 96-well microplates (Figs. 2 and 3). The CPE appearance ratio was 50.6% at 1 TCID_50_ adsorption (60 particles), and infection efficiency/particle was calculated as 0.84%. Compared to this, 7.7 TCID_50_ (7.7 × 60 = 462 particles) adsorption was required to induce 100% CPE; therefore, the infection efficiency/particle was calculated as 0.22%, which was significantly lower than that at 1 TCID_50_ adsorption (0.84%). Thus, the infection efficiency of NNV particles adsorbed onto cells is not constant. Using the semilogarithmic graph, we also proved that CPE appearance ratio reduced at a constant rate each time the NNV infectious dose was halved (Fig. 1B). This indicates that the CPE appearance ratio and viral infectious dose are not proportional. If they are proportional, halving of NNV infectious dose should also halve the CPE appearance ratio, as shown by the following regression curve: *y* = *50 e *^*2.3x*^ (Fig. S2A).

**Figure 5:**
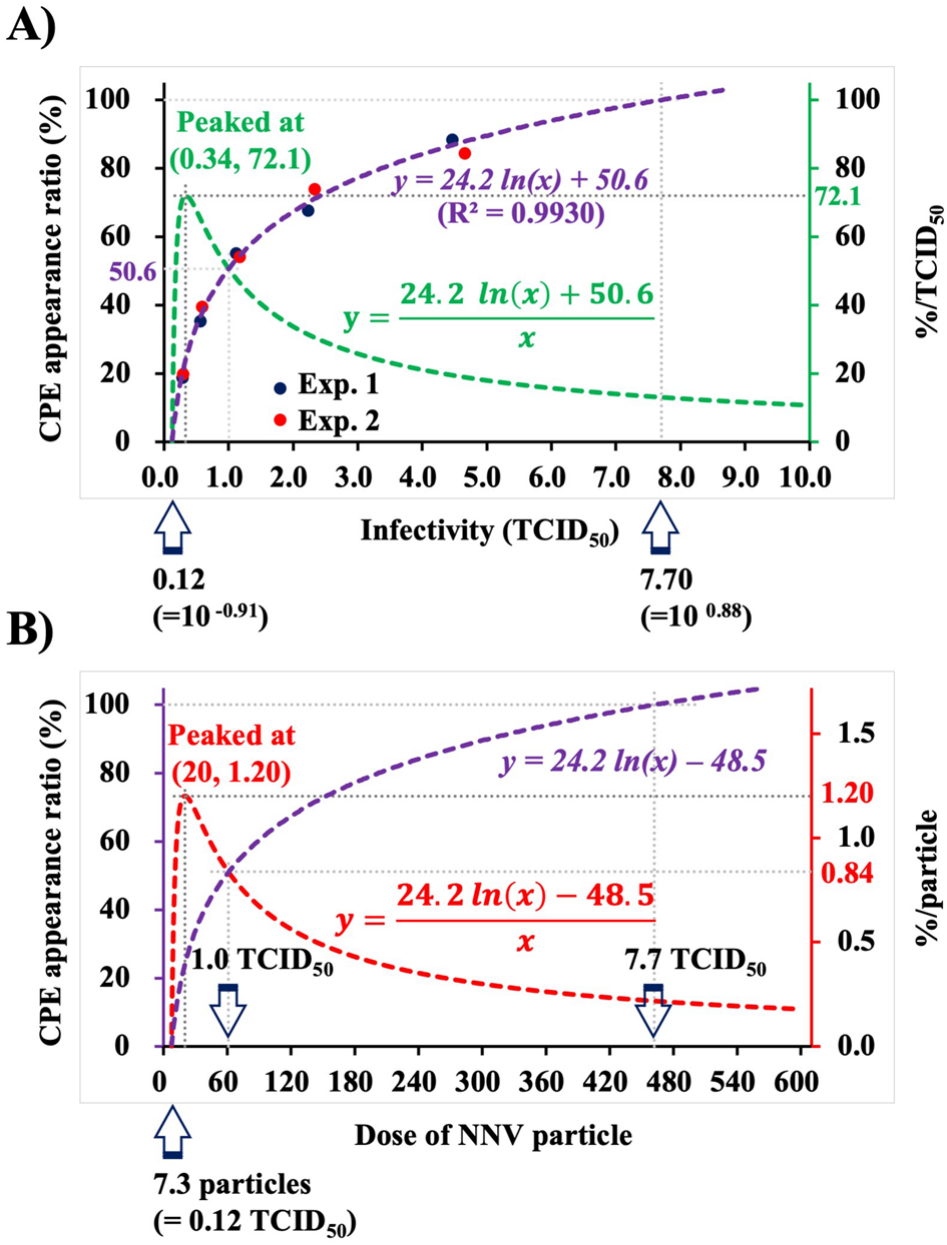
Alteration of infection efficiency of NNV particles adsorbed onto cells. (**A**) Graph showing CPE appearance ratio (%) on the 1st Y axis, infection efficiency/TCID_50_ (%/TCID_50_) on the 2nd Y axis, and infectious dose (TCID_50_) on the X axis. The blue broken line is a regression curve (*y* = *24.2 ln[x]* + *50.6*), which indicates that each time NNV infectious dose is halved, CPE appearance ratio reduces by 17%. The data shown in Fig. 1B are replotted to this graph. The green broken curve indicates infection efficiency/NNV infectious dose (%/TCID_50_), which was estimated as follows: *y* = *(24.2 ln[x]* + *50.6)/x* of NNV infectivity. Infection efficiency of NNV per TCID_50_ (%/TCID_50_) peaked at 0.34 TCID_50_ (72.1%/TCID_50_). (**B**) Graph showing CPE appearance ratio (%) on the 1st Y axis, %/particle on the 2nd Y axis, and NNV particle dose on the X axis. The data shown in (**A**) were re-evaluated based on the results that 1 TCID_50_ contains 60 particles and replotted to this graph. The red broken curve indicates infection efficiency/NNV particle (%/particle), which was calculated as follows: *y* = *(24.2 ln[x] − 48.5)/x* of NNV particle dose. Infection efficiency/NNV particle (%/particle) peaked at 20 particles (1.18%/particle). NNV, nervous necrosis virus; CPE, cytopathic effect; TCID_50_, 50% tissue culture infectious dose.

To predict alteration of NNV infection efficiency, the data of the semilogarithmic graph (Fig. 1B) were replotted on a normal graph, which yielded a new regression curve as follows: *y* = *24.2 ln(x)* + *50.6,* R^2^ = 0.9930 (Fig. 5A), thus showing that the increase in CPE appearance ratio gradually slows down with increasing NNV infectivity. Moreover, dividing *y* = *24.2 ln(x)* + *50.6* with NNV infectivity value yielded a new curve represented by the following function: *y* = *(24.2 ln [x]* + *50.6)/x* (Fig. 5A, green broken line). This curve indicates that CPE appearance ratio is altered with change in NNV infectious dose. CPE appearance ratio increased drastically with increasing NNV infectivity from 0.12 TCID_50_, which peaked at 0.34 TCID_50_, and gradually decreased thereafter. CPE appearance ratio/NNV infectious dose at the peak was calculated as 72.1%/TCID_50_. Subsequently, CPE appearance ratio/particle was calculated based on the result that 1 TCID_50_ contained 60 particles. The function *“y* = *(24.2 ln[x] − 48.5)/x*” indicated alteration of CPE appearance ratio/NNV particle (Fig. 5B), which increased drastically with increasing NNV dose from 7.3 particles, peaked at 20 particles, and gradually decreased thereafter. CPE appearance ratio/NNV dose at the peak was calculated as 1.20%/particle. Based on these results, we concluded that NNV infection efficiency is altered depending on the quantity of NNV particles adsorbed onto cells. Although the mechanism behind this was unclear, we believe that factors related to the host cell were involved.

Furthermore, alteration of CPE appearance ratio/NNV particle was calculated based on the following theoretical regression line: *y* = *50x* + *50* (Fig. S2A). This regression line, which passed through two points (i.e., 10 TCID_50_, 100% and 1 TCID_50_, 50%), showed that each time the NNV infectious dose was halved, the CPE appearance ratio linearly reduced at a constant rate. Moreover, based on the theoretical curve*,* NNV infection efficiency/TCID_50_ in vitro peaked at 0.27 TCID_50_ (79.9%/TCID_50,_ Fig. S2B). In our previous pathogenicity studies using an individual rearing system to prevent horizontal transmission, fish mortality was found to increase linearly with increasing infectious NNV dose (log_10_ TCID_50_)^16^, which is similar to the relationship between CPE appearance ratio and infectious dose in this study*.* Thus, alteration of NNV infection efficiency is also likely to occur in vivo depending on viral dose.

In conclusion, this study demonstrated that subtle differences in NNV infectivity can be accurately detected using the 96-well titration system. The CPE appearance ratio reduced by 17% each time the NNV infectivity dose was halved. NNV suspension at 1 TCID_50_ contained 60 particles, and almost all the NNV particles introduced into 96-well microplates as suspensions in ≤ 100 µl got adsorbed onto cells within 4 days of incubation. When these values were mathematically analyzed, the maximum CPE appearance ratio/NNV particle was calculated as 1.20%/particle. However, infection efficiency of NNV may alter depending on the quantity of viral particles adsorbed onto cells. In our previous studies regarding the mechanism of NNV cell adsorption and infection^12,13,20,22^, detecting subtle differences in virus infectivity titers was extremely challenging. This problem was solved in the present study; therefore, the mechanisms of NNV adsorption and infection may be better understood.

## Methods

### Viruses

NNV (SgNag05 isolate, RGNNV genotype, serotype C)^17,18^ was cultured using SSN-1 cells^5^ maintained at 25 °C with Leibovitz’s L-15 medium (Gibco) supplemented with 10% (v/v) fetal bovine serum (FBS) (Gibco) and 100 U/ml penicillin–streptomycin solution (Gibco) (L-15_10_ medium). NNV particles were purified by methods described previously^19–22^. Cultured NNV suspension was centrifuged at 12,000 × *g* for 20 min at 4 °C. The resultant supernatant was dialyzed using a Biotech cellulose ester (CE) membrane tube with a molecular weight cut off (MWCO) of 10^6^ (Spectrum Laboratories). After anion-exchange chromatography with a Hi-trap Q column (GE Healthcare), NNV particles were eluted with 700 mM NaCl. The resultant NNV suspension was desalted and concentrated by centrifugal ultrafiltration using a membrane with MWCO of 10^5^ (Vivaspin, Sartorius).

### Titration of NNV infectivity

NNV infectivity titers were determined using 96-well microplates seeded with SSN-1 cells. Appearance of CPE was evaluated after 2 weeks of culturing at 25 °C to determine TCID_50_. When NNV infectivity is high (> 10^2.0^ TCID_50_/ml), the sample is diluted serially 10 times, and 50 µl of each dilution sample is inoculated into 4 wells; this is the commonly used 4-well titration system. TCID_50_ values were calculated using the following numerical formula based on Kärber’s method^15^:

$${\text{log}}_{10} {\text{ TCID}}_{50} = {\text{D}} + {\text{I}} \times \left( {\sum \frac{{{\text{r}}_{i} }}{{{\text{n}}_{i} }} + 0.5} \right)$$

Here, D is log_10_ of the reciprocal of the highest dilution showing 100% CPE; I**,** log_10_ of the dilution factor; n_i_, number of wells used for each dilution; and r_i_, number of wells among n_i_ that were CPE-positive.

In contrast, the 96-well titration system is used for NNV suspension with low infectivity (≤ 10^2.0^ TCID_50_/ml). In this case, the sample was diluted serially twice, and each dilution sample was inoculated into 96 wells at the rate of 100 µl/well. The infectivity titers in this system were determined based on the standardized regression line obtained in Fig. 1B.

### Electrophoresis

Before performing SDS − PAGE, NNV particles were suspended in SDS-denature buffer (2% [w/v] SDS, 0.05% [v/v] ß-mercaptoethanol in 25 mM Tris-HCl [pH 6.8]) and boiled for 3 min. After cooling, tracking dye and glycerol mixture (0.5% [w/v] bromophenol blue and 70% [v/v] glycerol in 25 mM Tris-HCl [pH 6.8]) were added to each denatured sample. SDS − PAGE was performed under reducing conditions according to Laemmli’s method^23^ using 10% polyacrylamide gel. Gel proteins were stained with CBB (R-250, Wako). Density profiles for digital images of NNV CP, BSA and molecular weight markers in SDS–polyacrylamide gel were analyzed using ImageJ software version 1.53 (National Institutes of Health, Bethesda, Maryland, USA)^24^.

### Statistical analysis

Infectivity titers of NNV and sizes of SSN-1 cells were analyzed by t-test using Microsoft Excel software version 16.59 (Microsoft Corporation, USA). Statistical significance level was set at *p* < 0.05.

## Supplementary Information

Supplementary Figures.

## Data Availability

The datasets generated and/or analyzed during the current study are available from the corresponding author on reasonable request.
