# Supplementary material for: In vitro infection efficiency of nervous necrosis virus alters depending on amount of viral particles adsorbed onto cells
Source: Sci Rep. 2023 Jul 29;13:12305. doi: 10.1038/s41598-023-39426-6 (PMC10387107; doi:10.1038/s41598-023-39426-6)
Supplement: Supplementary file 1 — Supplementary Figures. [file 41598_2023_39426_MOESM1_ESM.pdf]

In vitro infection efficiency of nervous necrosis virus alters depending on amount of viral particles adsorbed onto cells

Han Sol Lee, Hyun Jung Gye, Toyohiko Nishizawa

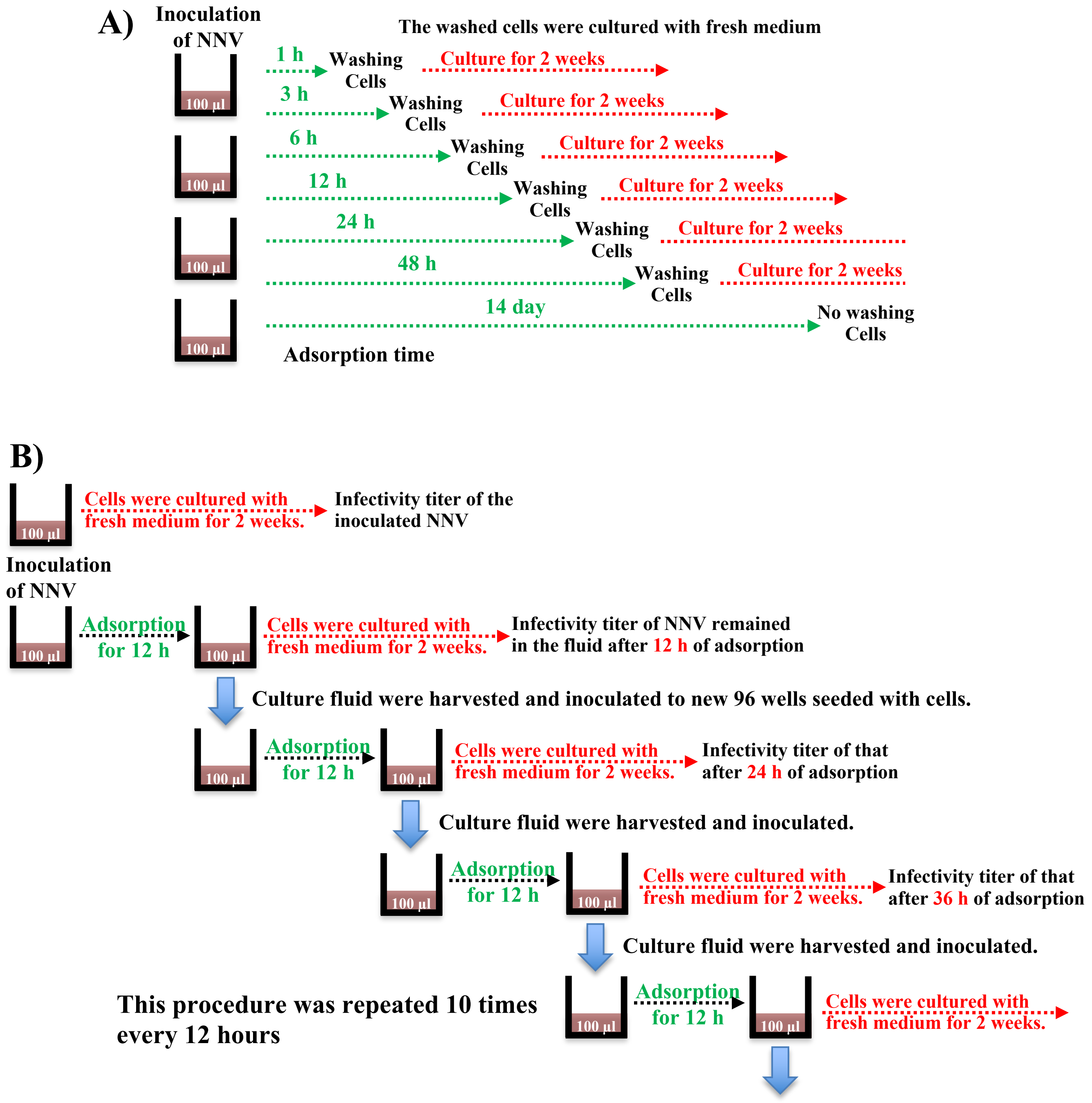

Supplementary Figure S1. Schematic illustration for the experiment procedures on correlation between CPE appearance ratio and NNV adsorption time.
A) Alteration of CPE appearance ratio depending on NNV-adsorption time (Fig. 3A), B) infectivity alteration of NNV that remained in culture supernatant depending on NNV adsorption time (Fig. 3B).
NNV, nervous necrosis virus; CPE, cytopathic effect

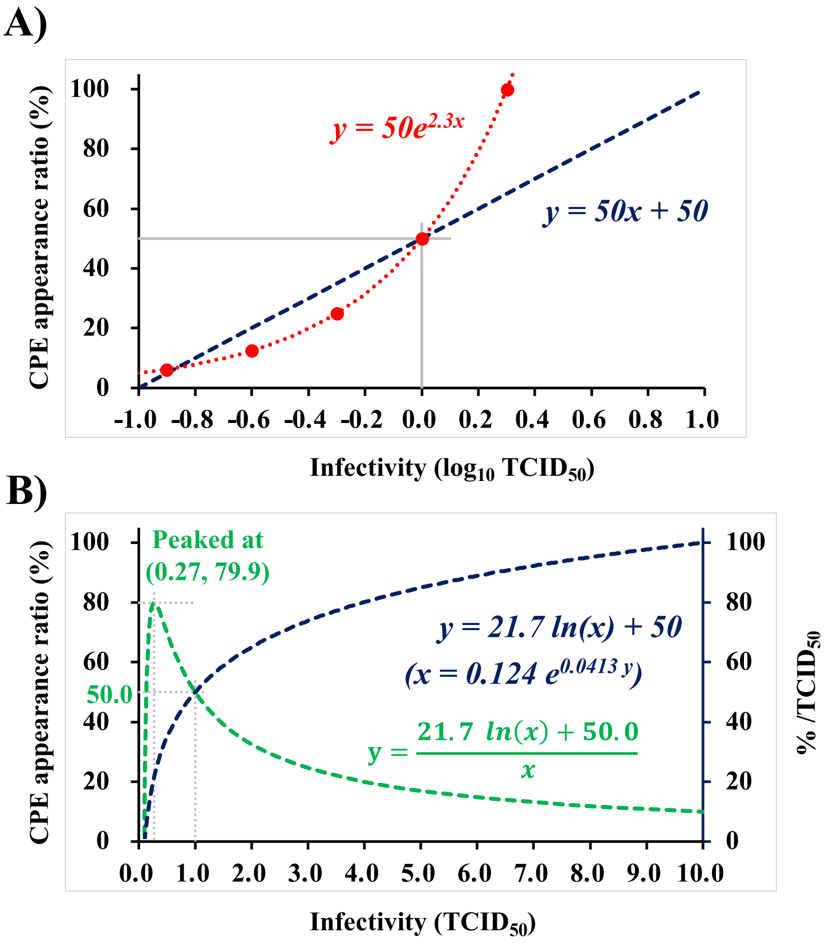

Supplementary Figure S2. Theoretical infection efficiency of NNV particles.
A) Semilogarithmic graph with CPE appearance ratio (%) on the Y axis and infectious dose ($\log_{10}$ $TCID_{50}$) on the X axis. The blue broken regression line indicates a theoretical regression line ($y = 50x + 50$). This regression line passes through two points (10 $TCID_{50}$, 100% and 1 $TCID_{50}$, 50%) and indicates that each time NNV infectious dose is halved, CPE appearance ratio reduces linearly at a constant rate. The red broken line indicates a theoretical regression curve ($y = 50\ e^{2.3x}$). This regression curve passes through a single point (1 $TCID_{50}$, 50%) and indicates that each time NNV infectious dose is halved, CPE appearance ratio also reduces by half. B) Normal graph with CPE appearance ratio (%) on the 1st Y axis, %/infectious dose (%/$TCID_{50}$) on the 2nd Y axis, and infectious dose ($TCID_{50}$) on the X axis. The blue broken regression line indicates a theoretical regression curve ($y = 21.7\ ln[x] + 50$), corresponding to the $y = 50x + 50$ regression line showed in Fig. S1A. The green broken curve indicates alteration of infection efficiency depending on NNV infectious dose (%/$TCID_{50}$), which was estimated as follows: $y = (21.7\ ln[x] + 50)/x$ of NNV infectivity. Theoretical infection efficiency/NNV infectious dose (%/$TCID_{50}$) peaked at 0.27 $TCID_{50}$ (79.9%/$TCID_{50}$). NNV, nervous necrosis virus; CPE, cytopathic effect; $TCID_{50}$, 50% tissue culture infectious dose

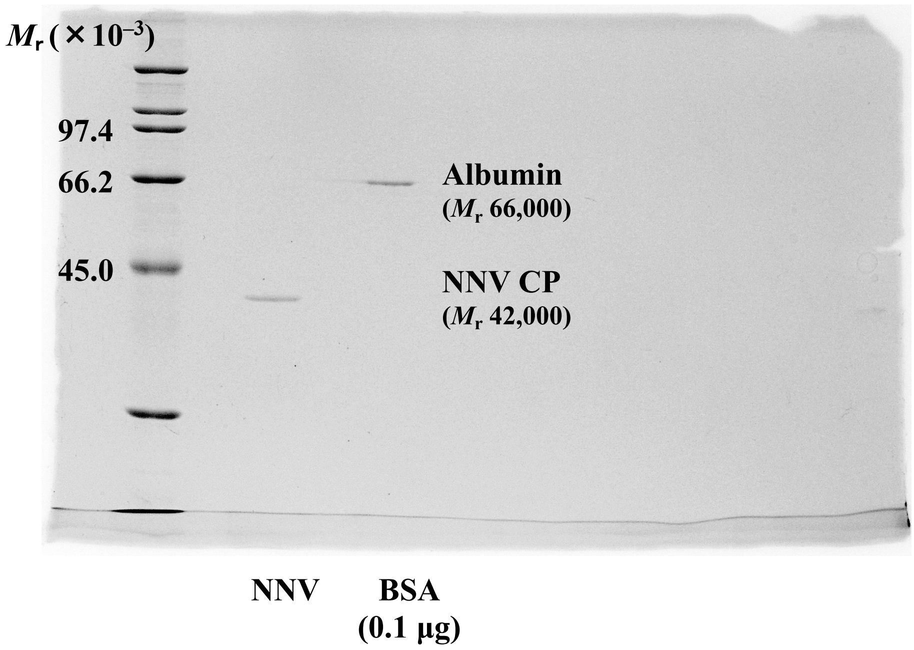

Supplementary Figure S3. An untrimmed photograph of the SDS-polyacrylamide gel shown in Fig. 4B.
